# Fluid-structure interaction in abdominal aortic aneurysms: effects of asymmetry and wall thickness

**DOI:** 10.1186/1475-925X-4-64

**Published:** 2005-11-04

**Authors:** Christine M Scotti, Alexander D Shkolnik, Satish C Muluk, Ender A Finol

**Affiliations:** 1 Biomedical Engineering Department, Carnegie Mellon University, Pittsburgh, Pennsylvania, USA; 2 Department of Mathematical Sciences, Carnegie Mellon University, Pittsburgh, Pennsylvania, USA; 3 Division of Vascular Surgery, Allegheny General Hospital, Pittsburgh, Pennsylvania, USA; 4 Institute for Complex Engineered Systems and Biomedical Engineering Department, Carnegie Mellon University, Pittsburgh, Pennsylvania, USA

## Abstract

**Background:**

Abdominal aortic aneurysm (AAA) is a prevalent disease which is of significant concern because of the morbidity associated with the continuing expansion of the abdominal aorta and its ultimate rupture. The transient interaction between blood flow and the wall contributes to wall stress which, if it exceeds the failure strength of the dilated arterial wall, will lead to aneurysm rupture. Utilizing a computational approach, the biomechanical environment of virtual AAAs can be evaluated to study the affects of asymmetry and wall thickness on this stress, two parameters that contribute to increased risk of aneurysm rupture.

**Methods:**

Ten virtual aneurysm models were created with five different asymmetry parameters ranging from β = 0.2 to 1.0 and either a uniform or variable wall thickness to study the flow and wall dynamics by means of fully coupled fluid-structure interaction (FSI) analyses. The AAA wall was designed to have a (i) uniform 1.5 mm thickness or (ii) variable thickness ranging from 0.5 – 1.5 mm extruded normally from the boundary surface of the lumen. These models were meshed with linear hexahedral elements, imported into a commercial finite element code and analyzed under transient flow conditions. The method proposed was then compared with traditional computational solid stress techniques on the basis of peak wall stress predictions and cost of computational effort.

**Results:**

The results provide quantitative predictions of flow patterns and wall mechanics as well as the effects of aneurysm asymmetry and wall thickness heterogeneity on the estimation of peak wall stress. These parameters affect the magnitude and distribution of Von Mises stresses; varying wall thickness increases the maximum Von Mises stress by 4 times its uniform thickness counterpart. A pre-peak systole retrograde flow was observed in the AAA sac for all models, which is due to the elastic energy stored in the compliant arterial wall and the expansion force of the artery during systole.

**Conclusion:**

Both wall thickness and geometry asymmetry affect the stress exhibited by a virtual AAA. Our results suggest that an asymmetric AAA with regional variations in wall thickness would be exposed to higher mechanical stresses and an increased risk of rupture than a more fusiform AAA with uniform wall thickness. Therefore, it is important to accurately reproduce vessel geometry and wall thickness in computational predictions of AAA biomechanics.

## Background

Abdominal aortic aneurysms (AAA) are local enlargements of the aorta that occur preferentially below the renal bifurcation and they represent a socially relevant cardiovascular health disease. A recent study [1] reports that the prevalence of AAA disease is 8.8% in the population above 65 years of age and men are affected more often than women by a ratio of 4:1 [2]. Aneurysms are little-known among the lay public, but they are a significant cause of mortality; fifteen thousand people per year die from AAA rupture in the United States alone, making it the 13^th ^leading cause of death in this country and affecting 1 in 250 individuals over 50 years of age. Since the likelihood of being diagnosed with an aneurysm increases with age, the incidence of aortic aneurysmal disease is expected to increase with the continuously aging population. Aneurysms can be treated surgically; however typical treatment is based on the surgeon's estimation of the risk of rupture and the patient's general fitness for surgery, along with his/her life expectancy. Despite significant improvements in surgical procedures and imaging techniques, the mortality and morbidity rates associated with untreated ruptured AAAs remain very high. AAA disease is a health risk of considerable importance since this kind of aneurysm is mostly asymptomatic until its rupture, which is frequently a lethal event with an overall mortality rate in the 80% to 90% range [3]. The optimal strategy is clear: prevention of aneurysm rupture is the primary goal in management of aneurysmal disease.

Deciding between elective aneurysm repair and conservative management of the disease is difficult due to the lack of a reliable predictor of rupture risk. A critical AAA transverse diameter of 5 to 6 cm is the most common threshold value used clinically to recommend surgical repair or endovascular intervention [4,5]. However, small aneurysms can also rupture and the overall mortality associated with these may exceed 50% [6]. Therefore, ideally, the decision to repair an aneurysm should not be guided by maximum transversal dimension alone, but rather by a more reliable criterion associated with the actual rupture potential of the patient-specific artery, such as peak AAA wall stress and strength [7]. Since aneurysm rupture is a phenomenon that occurs when the mechanical stress acting on the dilating inner wall exceeds its failure strength, a criterion for repair based upon quantifying aneurysm stress and strength could facilitate a better method to determine at-risk AAAs. Unfortunately, there is no current method of obtaining *in vivo *measurements of tissue stresses or strength. However, mathematical and computational models can be utilized to predict the fluid and solid mechanics environments within aneurysmal aortas.

AAA wall stress is the outcome of several factors, such as characterization of the wall material, the shape and size of the aneurysm sac, the presence of intraluminal thrombus (ILT), and the dynamic interaction of the wall with blood flow. Since the internal mechanical forces are maintained by the dynamic action of blood flowing in the aorta, the quantification of the *hemodynamics *of AAAs is essential for the characterization of their biomechanical environment. The justification of biomechanics research is based on the well-known fact that both fluid and wall mechanics play an important role in pathologic conditions of blood vessels.

Prior works have examined the computationally predicted and experimentally validated flow patterns within virtual AAA models [8-11], showing the effect of aneurysm asymmetry on the increase in flow-induced wall pressure and wall shear stress. This has led to the use of patient-specific models obtained from diagnostic images that allow the prediction of flow-induced stresses on a single patient basis [12]. Similarly, Di Martino and colleagues [13] provided the notion of interaction between solid and fluid domains as it contributes to aneurysm rupture potential. This interaction between the domains was recently compared with a peak systolic static prediction of wall stresses in the presence of ILT [14]. Fully-coupled fluid-structure interaction (FSI) of the domains allows computation of the flow and pressure fields in the aneurysm, simultaneously with the wall stresses [15]. This methodology provides a method for validating the computational results with clinical diagnostic data, such as Echo Doppler flow visualization. Therefore, it is important to include both the dynamics of blood flow as well as the wall motion response associated with the pulsatile nature of the flow to accurately model the aneurysm. With the continuous improvements in computer architecture processing times, patient-specific computational models in a clinical setting are likely to be used in the near future as a tool for effective decision making in AAA surgical and endovascular repair.

In this work we describe the complex interaction of blood flow and the compliant AAA wall by utilizing a time dependent, fully-coupled FSI methodology to determine the effects of aneurysm asymmetry and wall thickness heterogeneity on the mechanical stresses and vortex dynamics. Ten virtual models were utilized in the study, which aims to provide a non-invasive methodology for quantifying transient AAA wall mechanics. The latter can then be compared with mean AAA wall tissue strength to provide a predictor for rupture potential. Additionally, the FSI technique is compared with quasi-static and transient solid stress analyses as alternative approaches for the reduction of computational processing time.

## Methods

### AAA geometry

**Figure 1:**
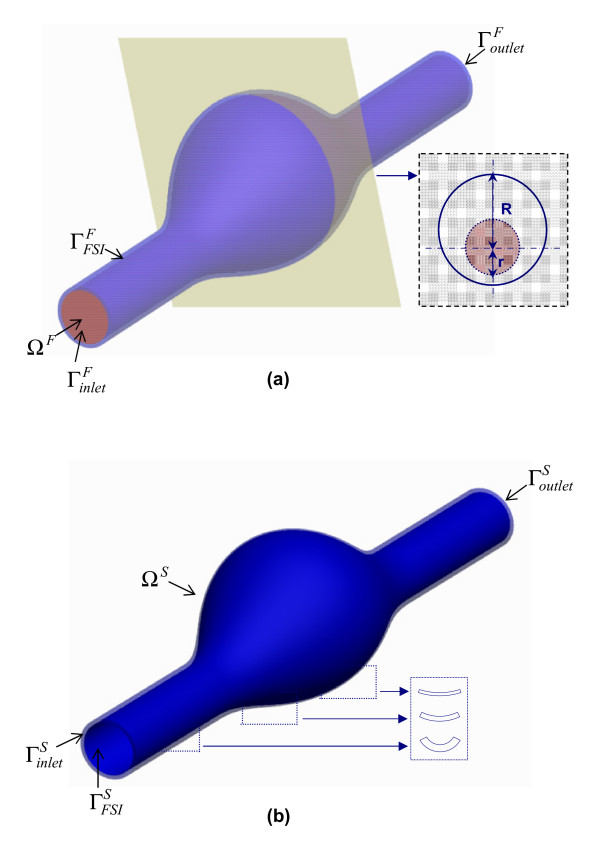
CAD geometry for β = 0.6 model: (a) fluid and solid domains with uniform wall thickness (b) solid domain with variable wall thickness.





Ten virtual aneurysm models were generated with the CAD software ProEngineer Wildfire (Parametric Technology Corporation, Needham, MA). The models differ in degree of asymmetry and wall heterogeneity, and are comprised by a fluid domain, Ω^F^, representing the aortic lumen and a solid domain, Ω^S^, representing the AAA wall. The fluid domain is characterized by circular cross sections parallel to the x-y plane, with an undilated diameter, d = 2 cm, at the inlet and outlet sections and a maximum diameter, D = 3 d, at the midsection of the AAA sac. The asymmetry of the model is governed by the β parameter given by Eq. (1).



where r and R are the radii measured at the midsection of the AAA sac from the longitudinal z-axis to the posterior and anterior walls, respectively, as shown in the inset of Figure 1(a). Thus, β = 1.0 yields an axisymmetric aneurysm. The geometry of the fluid domain is given by Eq. (2), which defines the diameter of each cross section, φ(z), and the deviation of its centroid from the z-axis, δ(z):

The geometry of the solid domain is given by an AAA wall with either a (i) uniform thickness (UW) or a (ii) variable thickness (VW). Both types of wall designs model the thickness of the aneurysm as material extruding normally from the surface enclosing the lumen. The UW model has a thickness given by λ = 1.5 mm, while the VW model is given by λ(z) = 1.5d/φ(z) (in mm) at each cross-section. It follows that the local wall thickness varies between 0.5 mm and 1.5 mm (with a mean of 1.0 mm for the AAA sac), inversely proportional to the local diameter of the cross-section. For a ruptured AAA, wall thickness can be as low as 0.23 mm at the rupture site with a surface-wide average of 1.45 mm [16]. The asymmetry of a virtual AAA is given by βε {1.0, 0.8, 0.6, 0.4, 0.2}, thus yielding a total of ten geometries, five of which have a uniform wall thickness (UW) and the other five variable wall thickness (VW). β = 1.0 corresponds to azymuthal symmetry, and β = 0.2 is an AAA for which only the anterior wall is dilated while the posterior wall is nearly flat. Figure 1(a) shows β = 0.6 with fluid domain Ω^F ^entities in a uniform thickness model. Figure 1(b) illustrates qualitatively the variable thickness domain Ω^S ^at three different wall locations for the same β = 0.6 model.

### Governing equations and boundary conditions

The governing equations for the fluid domain are the continuity and Navier-Stokes equations with the assumptions of homogenous, incompressible, and Newtonian flow. Since the fluid domain is deformable in an FSI problem, an Arbitrary Lagrangian-Eulerian (ALE) formulation has been adopted. The ALE formulation [17] introduces a moving coordinate system to model the deformation of the fluid domain. The momentum and mass conservation equations governing the flow are given by Eq. (3) in ALE form:



ρ_f_Δ·**v **= 0     (3b)

where ρ_f _is the fluid density, **τ**_f _is the fluid stress tensor,  are the body forces per unit volume, **v **is the fluid velocity vector, and  is the moving coordinate velocity, respectively. In the ALE formulation,  is the relative velocity of the fluid with respect to the moving coordinate velocity. Blood is modeled to have a density ρ_f _= 1.05 g/cm^3 ^and a dynamic viscosity μ = 3.85 cP. The governing equation for the solid domain is the momentum conservation equation given by Eq. (4). In contrast to the ALE formulation of the fluid equations, a Lagrangian coordinate system is adopted:



where ρ_s _is the AAA wall density, **τ**_s _is the solid stress tensor,  are the body forces per unit volume, and  is the local acceleration of the solid. The AAA wall is assumed to be an isotropic, linear, elastic solid with a density ρ_s _= 2.0 g/cm^3^, a Young's Modulus E = 2.7 MPa and a Poisson's ratio υ = 0.45. The wall material implemented in this work represents a tissue of average characteristics for the aneurysmal abdominal aorta, i.e. a linearization of the stress-strain curve as reported in [13]. Previous studies have shown that aneurysm tissue is a non-linear, isotropic, hyperelastic material [18]. Hence, the constitutive linearity of the AAA wall is a simplification of the FSI and stress analyses in this study. In the FSI formulation, the AAA wall is assumed to undergo large displacements and small strains.

**Figure 2:**
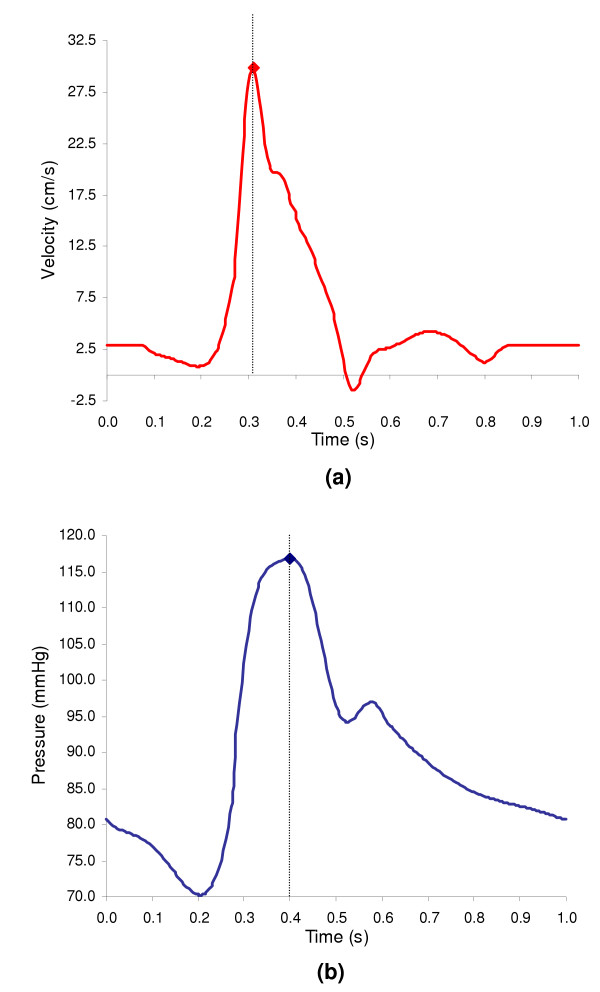
*In vivo *luminal pulsatile velocity and pressure reproduced from [19]: (a) velocity waveform (b) pressure waveform. Inlet peak systolic flow occurs at t = 0.304 s and outlet peak systolic pressure at t = 0.4 s.



The boundary of the fluid domain is divided into the following regions for the assignment of boundary conditions: inlet (), outlet (), and the fluid-structure interaction interface (), as shown in Figure 1(a). The applied boundary conditions on the non-FSI regions are (i) a time dependent fully developed velocity profile on  and (ii) a time dependent normal traction (due to luminal pressure) on . These are presented by Eq. (5) as follows:





where τ_nn _is the normal traction, u(t) and p(t) are the time dependent velocity and pressure waveforms shown in Figure 2,  designates the normal of the respective boundary, and **I **is the standard identity matrix. Time dependency, as introduced by u(t) and p(t), is given by Fourier series representations of the waveforms generalized in Eq. (6):

N is the number of harmonics used to reproduce the *in vivo *measurements of luminal velocity (N = 18), u(t), and pressure (N = 7), p(t), respectively. These waveforms are triphasic pulses appropriate for normal hemodynamics conditions in the infrarenal segment of the human abdominal aorta first reported by Mills et al [19]. The use of an input transient velocity based on normal physiology is justified by the fact that the inlet boundary condition is applied above the proximal neck of the aneurysm, an undilated segment of the abdominal aorta. For average resting conditions, blood flow in the abdominal aorta is generally laminar [20,21]; flow deceleration achieved after peak systole induces laminar disturbed flow conditions and vortex formation even under simulated exercise conditions [22-24]. Inlet peak systolic flow occurs at t = 0.304 seconds and outlet peak pressure at t = 0.4 seconds. The time-average Reynolds number is Re_m _= 410, which is characteristic of a patient in resting conditions [25]. Re_m _is calculated as , where  is the time-averaged, mean inlet velocity and d is the inlet diameter. The Womersley number, , characterizes the flow frequency ω (ω = 2π/T and T = 1.0 seconds), the geometry and the fluid viscous properties, and is α = 13.1, a typical value for the human abdominal aorta under resting conditions [26]. The amplitude coefficient of the velocity waveform is defined as γ = Re_peak_/Re_m _= 5.25.

Figure 1(b) shows the boundary of the solid domain divided into inlet (), outlet () and the fluid-structure interface () regions. The FSI interfaces  and  are identical, coupling the fluid and solid domains. The boundary conditions on the non-FSI regions of the solid domain impose zero translation on the ends  and  as given by Eq. (7). This corresponds to completely fixing the ends of the domain, simulating the tethering of the aorta by the surrounding tissue and organs. Numerical experimentation with the objective of minimizing the stresses at the proximal and distal necks yielded placement of the inlet and outlet sections at a distance 1.5d apart from the aneurysm sac.



The boundary condition at the outer wall surface corresponds to a reference zero normal traction, as the peritoneum and surrounding tissues do not exert any significant pressure on the arterial wall. There are no published data on normal forces exerted by internal organs and tissue on the wall abdominal aorta. The final set of boundary conditions is applied to the FSI interfaces  and  as follows: (i) displacements of the fluid and solid domain FSI boundaries must be compatible, (ii) tractions at these boundaries must be at equilibrium and (iii) fluid obeys the no-slip condition. These conditions are given by Eq. (8):







where **d**, **τ**, and  are displacement vectors, stress tensors, and boundary normals with the subscripts *s *and *f *indicating a property of the fluid and solid, respectively.

In addition to the FSI methodology, we investigated alternative computational solid stress (CSS) techniques for approximating the AAA wall stresses. In these analyses we disregard the blood flow and attempt to obtain comparatively accurate results by applying a spatially-uniform pressure function onto the inner wall. The CSS_T _method (transient CSS) applies the transient function p(t) from Eq. 4(b) to simulate the effect of luminal pressure acting on the inner wall. Similarly, the CSS_S _method (static CSS) applies p(t = 0.4) in a quasi-static formulation to obtain the stresses at peak systolic pressure. In these two approaches we utilize only the solid domain Ω^S ^as shown in Figure 1(b), with prescribed zero translation at the proximal and distal ends as given by Eq. (7), and with a pressure boundary condition as given by Eq.(9):



### Numerical discretization

**Figure 3:**
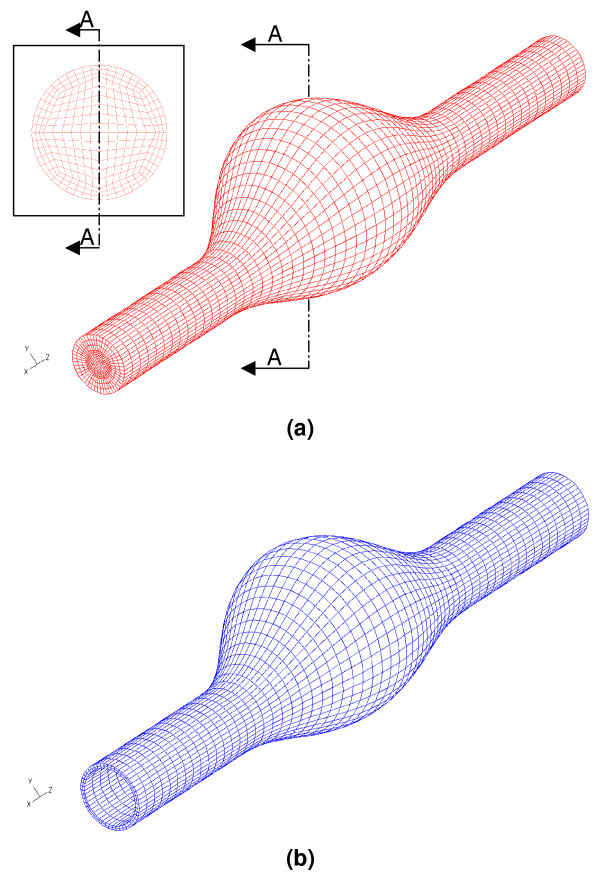
Computational domain for β = 0.6 models: (a) fluid mesh (b) solid mesh with variable wall thickness.

The software Adina (v8.0, ADINA R&D, Inc., Cambridge, MA) was utilized for the numerical simulation of fluid-structure interaction (FSI) between the wall and the lumen, as well as the alternative CSS_T _and CSS_S _analyses involving only the aneurysmal wall, as described in [15]. The Finite Element Method (FEM) is used to solve the governing equations, which discretizes the computational domain into finite elements that are interconnected by nodal points. In this work we make use of linear hexahedral, eight-node elements to discretize the fluid and solid domains. The mesh generator Gridgen (Pointwise Inc., Fort Worth, TX) is used to develop the finite element grids. The ten aneurysm models are all composed of 17,280 hexahedral elements (19,093 nodes) for the fluid and 5,760 hexahedral elements (8,784 nodes) for the solid domains. Figure 3 illustrates the fluid and solid meshes for β = 0.6. Mesh sensitivity analyses were conducted with four additional mesh sizes ranging from 12,480 to 44,928 fluid elements and 3,840 to 13,824 solid elements. Independence in mesh size was obtained for the primary variables (velocity components, fluid pressure and structural displacements) within 5% relative error for the 4^th ^mesh (32,256 fluid and 10,752 solid elements). However, the mesh used in the present study was chosen due to its adequate compromise between acceptable CPU simulation times (71 CPU-hours on average) and moderate relative errors of the primary variables at randomly selected nodal points (15% on average). In this regard, it is important to understand that this work is a baseline study for the development of a computational, pre-operative planning tool for physicians, and as such treatment decisions must be made within a reasonable turnaround time. Additionally, mass conservation in the fluid domain was met for a relative error (comparison between volume flow rate at the inlet and outlet sections, and rate of change of volume within the geometry) of 1%.

Equations (3–4) are reduced to weak form by following the standard Galerkin procedure [27]. In short, the fluid domain employs special *Flow-Condition-Based-Interpolation *(FCBI) hexahedral elements which use constant functions to interpolate velocity and bi-linear functions to interpolate pressure and displacements on each fluid element. The solid domain employs *Mixed-Interpolation *hexahedral elements, preferred in modeling nearly incompressible media, which use constant functions to interpolate pressure and bilinear functions to interpolate displacements on each solid element. The discretized equations for the fluid and solid elements are assembled into one system of equations, coupling the fluid and solid meshes. A sparse matrix solver based on Gaussian elimination is used for solving this system.

**Figure 4:**
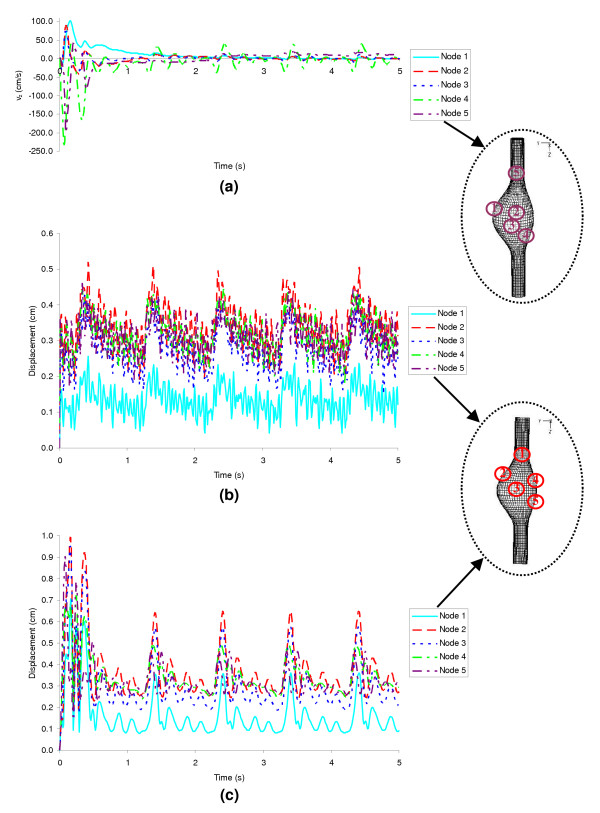
Time periodic convergence plot for β = 0.2: (a) velocity in the z-direction, (b) displacement for the FSI analysis and (c) displacement for the CSS_T _analysis. The insets show a schematic of the location of the nodal points used for the time convergence studies.

The FSI methodology utilizes an implicit time integration scheme, first applied on the fluid-structure interface of the fluid domain () where the coordinate system of the fluid and solid domains is Lagrangian. The results are then utilized to solve each domain entirely. Pulsatile flow is simulated over five to eight cycles with a time step size Δt_FSI _= 5·10^-3^seconds until periodic convergence is achieved. Figure 4 shows convergence for the fluid domain (4a) and the solid domain (4b) for β = 0.2 in terms of five nodal point values of axial velocity and displacement magnitude, respectively. For the purpose of comparison, Figure 4(c) illustrates the convergence of displacement magnitude for the CSS_T _analysis at the same five selected nodal points. The simulations were performed on a Tru64 Unix operating system using up to eight 1.15 GHz EV7 processors and in-memory computing. The CSS approaches only utilize the solid domain; hence, the final matrix assembly consists of only solid element equations and the computational times are significantly less when compared with the FSI computational times. For a consistent comparison, the CSS_T _system of equations is solved with the same solution methods as in FSI but with a time step size  seconds. The CSS_S _system of equations is solved with the sparse matrix solver for a steady state solution with a constant and uniform pressure p(t = 0.4) applied on the inner AAA wall. The computational times for the CSS_S _simulations are negligible in comparison with the CSS_T _(3 CPU-hours on average) and FSI (71 CPU-hours on average) simulations. A fully-coupled FSI method is computationally more expensive due to the memory required to adapt the matrices for a moving mesh algorithm and, thus, must be weighed against the clinical benefit of obtaining a complete characterization of the flow dynamics and wall stresses of the aneurysm sac.

## Results and discussion

### Asymmetry effect

**Figure 5:**
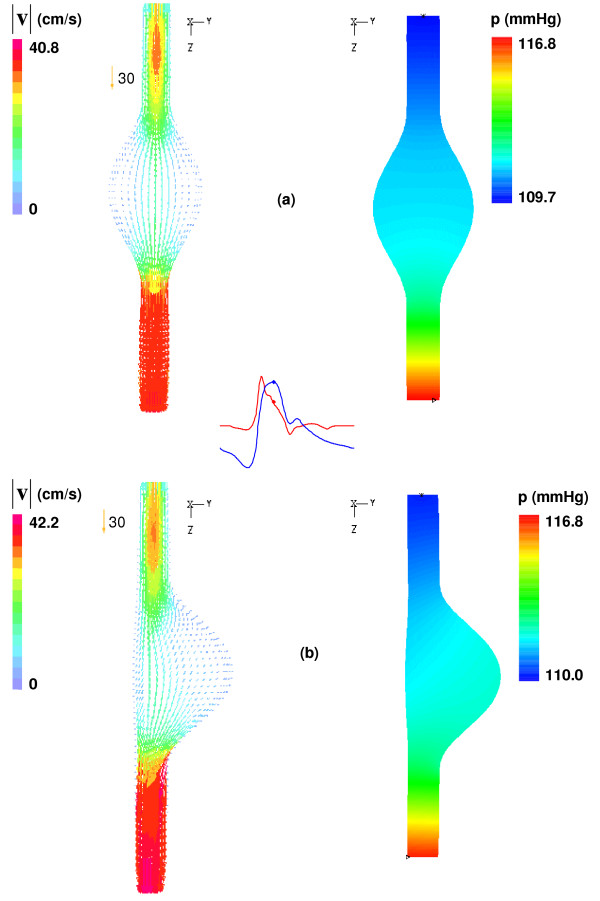
Velocity vectors and fluid pressure at the wall at t = 0.4 s in y-z plane for (a) β = 1.0 and (b) β = 0.2 uniform wall (UW) thickness models.

The blood flow dynamics in aneurysm models is governed by the relative compliance of the vessel, determined by its non-homogenous shape (asymmetry and wall thickness) and material characterization of the wall. Figure 5 shows the flow patterns for β = 0.2 and β = 1.0 at t = 0.4 s (peak systolic pressure in reference to the outlet normal traction boundary condition) for uniform wall thickness. The velocity vectors illustrate a streamlined profile absent of vortices, a flow path customarily associated with a condition of systolic acceleration. This flow characterization is significantly different from that described in [11] for the most asymmetric rigid AAA model (β = 0.3), since the energy stored by the expanding compliant vessel during systole ejects the vortex downstream shortly after peak flow. The phase delay (0.096 seconds) between the inlet velocity and outlet pressure waveforms also accounts for this difference, i.e. the flow has begun to temporally decelerate at the time the wall is fully expanded due to systolic pressure.

The wall pressure distribution between the two models is nearly identical at the longitudinal cross-section given by the YZ-plane. As the β = 0.2 model contains the same volume of fluid in the aneurysm sac this result is not entirely unexpected. Moreover, the reversal of the pressure gradient, given by a lower pressure at the inlet rather than at the outlet, signifies the previously stated effect of a phase shift on the flow dynamics, as well as the influence of the opposing pressure waveform imposed at the outlet. With the onset of diastolic flow conditions, the inlet velocity begins to decelerate, reducing the overall pressure gradient. As the cardiac cycle continues its course this pressure drop is expected to decrease, yielding flow reversal and recirculation regions dominated by convective effects, i.e. local acceleration due to bulging of the sac and asymmetry.

An assessment of the effect of asymmetry on flow patterns downstream of the AAA sac shows that for uniform wall thickness, the vortex dynamics generated for β = 0.2 and β = 1.0 is almost identical at the same temporal stages of the flow cycle. The vortices that develop and dissipate in the aneurysm, however, remain in the midsection to distal end of the sac for β = 1.0, whereas β = 0.2 is subject to stronger recirculating flows and the vortices travel upstream towards the proximal end of the aneurysm, particularly in diastole (t ≥ 0.6 s). This vortex translation along the anterior wall is the effect of retrograde flow caused by the velocity-pressure waveform phase shift and the instantaneous flow reversal experienced during diastole. The asymmetric geometry of the β = 0.2 model magnifies this effect, allowing for vortices to remain longer along the bulging anterior wall due to local flow deceleration. Conversely, the symmetric model has a reduced curvature along the anterior wall and the vortices develop and dissipate more readily as convective effects are weaker.

Vortex growth inside the AAA sac creates favorable conditions for increased platelet deposition rates, thrombus formation, and an increased risk of rupture [28]. The effect of aneurysm flow dynamics on the wall mechanics can be determined by predictions of Von Mises stress, an energetic formulation adopted in lieu of representing the nine components of the second order stress tensor. This is a stress quantity used in the field of failure mechanics, which characterizes the distortion energy (α σ^2^/E) of a material subject to loading and deformation. Therefore, it can be used as a criterion for failure with respect to an experimental, permissible stress value. According to the Huber-Von Mises-Hencky theory, failure is predicted to occur if Eq. (10) is valid:



where the left-hand-side term represents the square of the Von Mises stress, which is a function of the local principal stresses σ_1_, σ_2 _and σ_3 _at a particular state of stress of the structure, and σ_f _is the uniaxial failure strength of the material.

**Figure 6:**
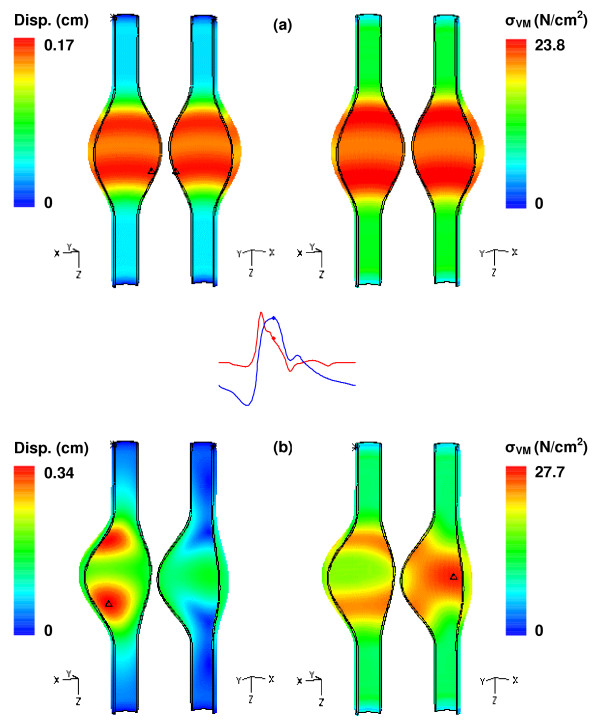
Displacement and Von Mises wall stress distributions at t = 0.4 s for (a) β = 1.0 and (b) β = 0.2 uniform wall (UW) thickness models. The symbol Δ indicates the location of the maximum wall stress.

Geometry has been well established as a contributing factor to aneurysm expansion and rupture potential, independently of the heterogeneity of the wall. Figure 6 shows the displacement magnitude and stress distributions for the β = 0.2 and β = 1.0 uniform wall thickness models. In each case significant gradients occur at the inflection points of the aneurysm curvature. For β = 1.0, the changes in curvature result in higher displacements and increased stress, suggestive of the effect of flow through the gradual expansions and contractions of the geometry. For β = 0.2, only the anterior wall (left frame) displays a displacement gradient at the inflection points, while the Von Mises stress is maximum at the posterior wall (right frame). This is the outcome of blood flow and fluid pressure acting on a wall of decreasing curvature, as the posterior wall of this model is nearly flat.

**Figure 7:**
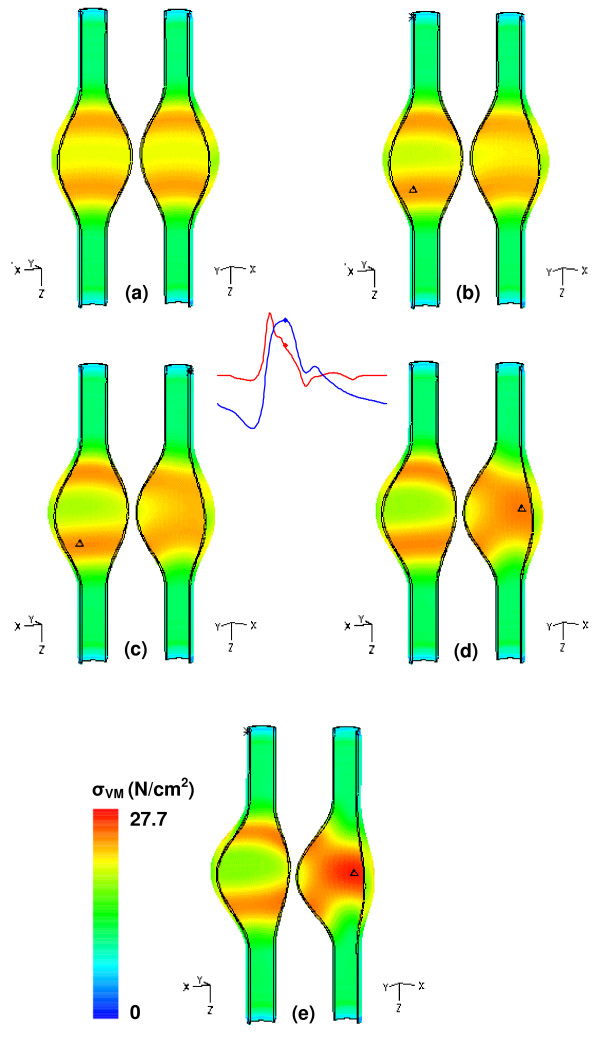
Von Mises wall stress distribution at t = 0.4 s for models of increasing asymmetry and uniform wall (UW) thickness: (a) β = 1.0, (b) β = 0.8, (c) β = 0.6, (d) β = 0.4, and (e) β = 0.2. The symbol Δ indicates the location of the maximum wall stress.

Comparing the magnitude of wall stress between β = 1.0 and β = 0.2 at t = 0.4 s reveals that a symmetric AAA is subject to a Von Mises stress 14% lower (23.8 N/cm^2 ^compared to 27.7 N/cm^2^) than a highly asymmetric one. The effect of asymmetry is confirmed by Figure 7, with the stress for all five models scaled to the maximum of the β = 0.2 model. The change in location of the maximum stress is due largely to the changing shape of the aneurysm sac. In particular, between β = 0.6 and β = 0.4, the location shifts from the anterior wall at the distal end to the posterior wall at the midsection, a consequence of the decreasing curvature of the posterior wall, which allows for high speed flow along this surface.

Previous authors have reported on the effect of asymmetric shape and geometry changes in AAA wall mechanics. Elger *et al *[29] studied axisymmetric hypothetical AAA models, concluding that circumferential stresses are larger for aneurysm shapes of smaller curvature at constant maximum diameter. In quasi-static solid stress analyses (CSS_S_), Vorp and associates [30] evaluated Von Mises stress distributions in virtual AAA models similar to those presented in this work, for varying asymmetry and varying maximum diameter. They report on a concomitant increase in wall stress with increasing diameter and asymmetry, with stress values of the same order of magnitude as those reported in the present FSI studies for the UW models.

### Wall thickness effect

A factor of increasing significance in AAA rupture risk prediction is the heterogeneity of the wall, in particular its thickness. It is difficult to accurately assess this dimension in patient-specific CT images due to calcification, thrombus, and the lack of clear image definition between the inner and outer wall surfaces. Therefore, a uniform thickness of 1.5 mm is typically assumed when modeling individual AAAs [13]. However, experimental sampling of wall specimens reveals that the wall is actually non-uniform, thinning in response to pulsatility and the progressive expansion of the aneurysm sac [31].

**Figure 8:**
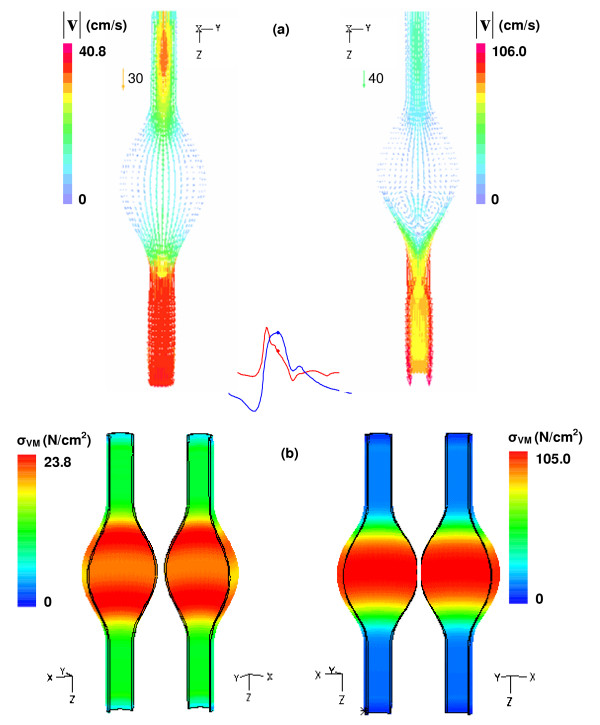
Effect of uniform (left) and variable (right) wall thickness: (a) y-z plane velocity vectors and (b) Von Mises stress distribution at t = 0.4 s for β = 1.0 model. The symbol Δ indicates the location of the maximum wall stress.

As evident from Figure 8, a variable wall thickness affects the flow dynamics as well as the stress distribution at t = 0.4 s, regardless of symmetry. For β = 1.0, a mostly attached flow pattern is evident with a uniform wall thickness, while a ring-shaped vortex is observed near the distal end for a variable wall thickness. Also of significance to the formation of vortices is the periodic nature of flow acceleration and retrograde flow found at the distal end in both models. In the variable wall thickness model the flow reverses direction more frequently, yielding negative flow rates at the distal end nearly twice as often compared to the uniform wall thickness model. This is mostly due to the increased compliance and larger deformation of the thinner wall that results from the momentum changes generated by the fluid throughout the cardiac cycle.

**Figure 9:**
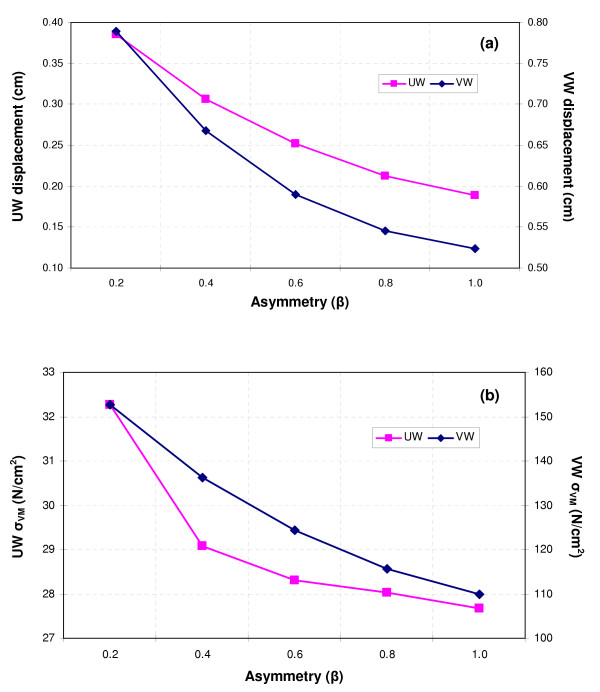
Effect of asymmetry on (a) peak displacement and (b) peak wall stress.

The patterns of Von Mises stress for the β = 1.0 model, shown in Figure 8 for t = 0.4 s, demonstrate the importance of wall thickness for a criterion of AAA rupture potential. The assumption of a uniform wall thickness translates into an underestimation of the maximum wall stress (23.8 N/cm^2 ^for UW and 105.0 N/cm^2 ^for VW) of nearly 77% when compared with a variable wall thickness model. The maximum stress occurs where the wall is thinnest, at the midsection of the sac; for the model with uniform wall thickness, the maximum stress occurs near the proximal and distal ends of the aneurysm, indicating the significance of geometry changes in aneurysm mechanics. This effect is illustrated in Figure 9 for both peak displacement and peak Von Mises stress, with the uniform wall scale represented by the left vertical axis and the variable wall scale according to the right vertical axis. For the FSI technique, peak wall stress and peak displacement are not achieved at t = 0.4 s as is the case for the CSS_S _and CSS_T _techniques, but rather during 0.304 s < t < 0.4 s corresponding to the phase delay interval between peak inlet and outlet boundary conditions. Therefore, it follows that the effects of flow and traction boundary conditions in FSI are not transmitted instantaneously to the AAA wall, which yields maximum deformation after inlet peak flow. This is due to the elastic energy stored at the AAA wall from the previous pulsatile cycles of the simulation leading up to and during the last pulsatile cycle, which is used for postprocessing purposes. In Figure 9, the variable wall thickness model shows elevated displacements and stresses, decreasing nonlinearly with increasing symmetry. Given a fixed asymmetry, the nonlinear variation in maximum wall stress due solely to the heterogeneous nature of the wall thickness is more significant: a 4-fold increase for β = 1.0 and 4.7-fold increase for β = 0.2. These results indicate that, independently of location, variable AAA wall thickness as hypothesized in this work has a more significant effect on the peak wall stress than the asymmetric shape of the aneurysm sac itself, for a fixed maximum transverse diameter. Further verification of the relative weight of these two variables in FSI AAA rupture risk prediction is required for patient-specific models.

### Comparison of computational methods

**Figure 10:**
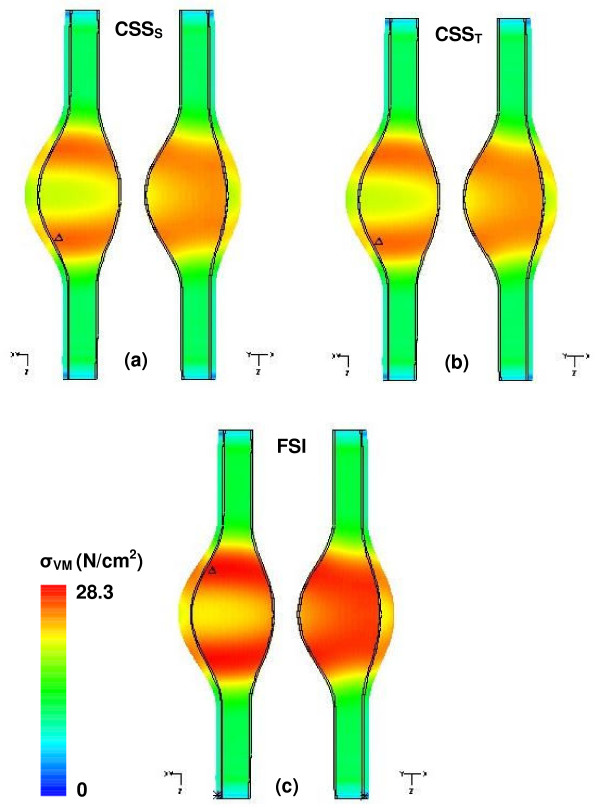
Comparison of peak Von Mises wall stress distributions for β = 0.6 UW model evaluated for: (a) static computational solid stress -CSS_S_- approaches, (b) transient computational solid stress -CSS_T_-, and (c) fluid-structure interaction -FSI. The symbol Δ indicates the location of the maximum wall stress.

The use of computational modeling and numerical techniques for the assessment of AAA rupture risk has been traditionally limited to the simulation of wall mechanics under quasi-static (CSS_S_) conditions [7,18]. This approach has the advantage of being able to model the wall with two-dimensional solid shell elements under the assumption of a uniform wall thickness while ignoring the fluid dynamic events within the aneurysm sac [29-35], which translates into a quick turnaround for the computation of peak wall stresses. We have tested our virtual AAA models using the CSS_S _technique, as well as for CSS_T _where the uniformly distributed luminal pressure is modeled as pulsatile and the maximum stresses evaluated at t = 0.4 s. Using our FSI-computed peak wall stresses as the baseline for comparison, Table 1 and Figure 10 (only for β = 0.6 and UW) show the difference in these stresses between the CSS_S_, CSS_T_, and FSI methodologies. From Table 1, under the assumption of a uniform wall (UW) thickness, the quasi-static solid stress computations result in an underestimation of the peak stress of 9.4% on average; similarly, the pulsatile solid stress technique underestimates the peak stress by 9.2% on average. Under the assumption of a heterogeneous wall thickness, the CSS_S _and CSS_T _techniques underestimate the peak stress predictions by an average of 29.5% and 29.4%, respectively. Given the importance of wall thickness heterogeneity in the accurate estimation of AAA rupture potential, these results indicate that fluid mechanics events should be taken into account in the modeling approach for the assessment of wall mechanics.

**Table 1:** Comparison of peak wall stress among the three numerical approaches.

**β**	**0.2**	**0.4**	**0.6**	**0.8**	**1.0**
**UW FSI**	32.3	29.1	28.3	28.0	27.7
**UW CSS_T_**	29.0 (-10.2)	26.3 (-9.6)	26.0 (-8.1)	25.6 (-8.6)	25.1 (-9.4)
**UW CSS_S_**	29.2 (-9.6)	26.3 (-9.6)	25.8 (-8.9)	25.4 (-9.3)	25.1 (-9.4)
**VW FSI**	152.6	136.2	124.3	115.6	110.0
**VW CSS_T_**	106.5 (-30.2)	95.7 (-29.7)	88.0 (-29.2)	82.3 (-28.8)	78.2 (-28.9)
**VW CSS_S_**	107.5 (-29.6)	95.6 (-29.8)	87.5 (-29.6)	82.0 (-29.1)	77.8 (-29.3)

Peak wall stress (in N/cm^2^) and comparison between the three numerical techniques. The parentheses show the % difference of the stress obtained with the CSS_T _and CSS_S _methods with respect to the baseline FSI method.

The virtual AAA models presented in this work provide a fundamental baseline for application of the FSI methodology as a non-invasive tool for rupture risk prediction in individual patients, outlining the importance of aneurysm asymmetry and non-uniformity of the vessel wall. This approach takes into account blood flow dynamics, which is inherently transient, and its effect on the wall mechanics. Hence, the results of the FSI predictions demonstrate the relationship between the fluid velocity field and the flow-induced wall stresses, which previous studies have assessed indirectly only on the basis of a uniform and static fluid pressure distribution. During the cardiac cycle, the instantaneous fluid forces acting on the inner wall will deform and expand the artery. In turn, the wall motion alters the velocity field until equilibrium is reached; these events occur instantaneously with the pulsating flow and cannot be evaluated by utilizing a CSS technique.

**Figure 11:**
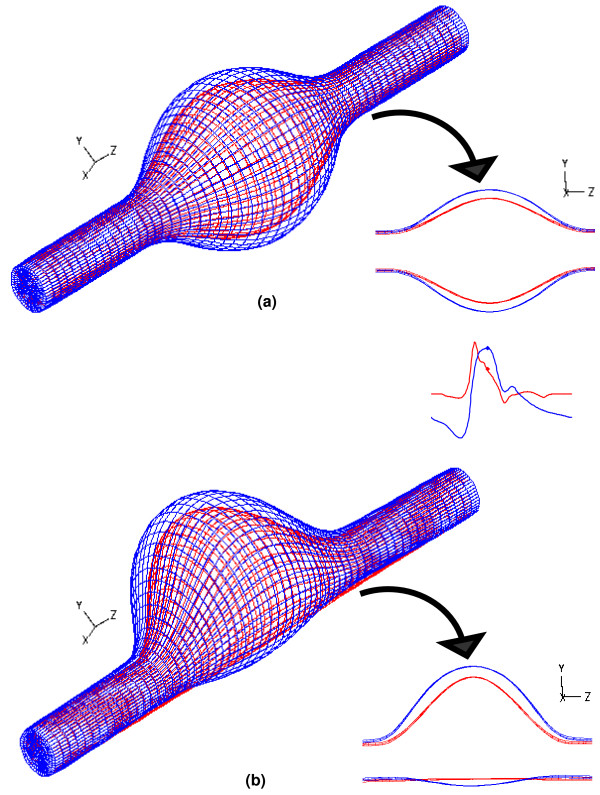
Deformation of the AAA sac at t = 0.4 s for (a) β = 1.0 and (b) β = 0.2 models with variable wall (VW) thickness. The red mesh is the original, non-deformed artery, while the blue mesh is the deformed geometry at peak systole.

**Figure 12:**
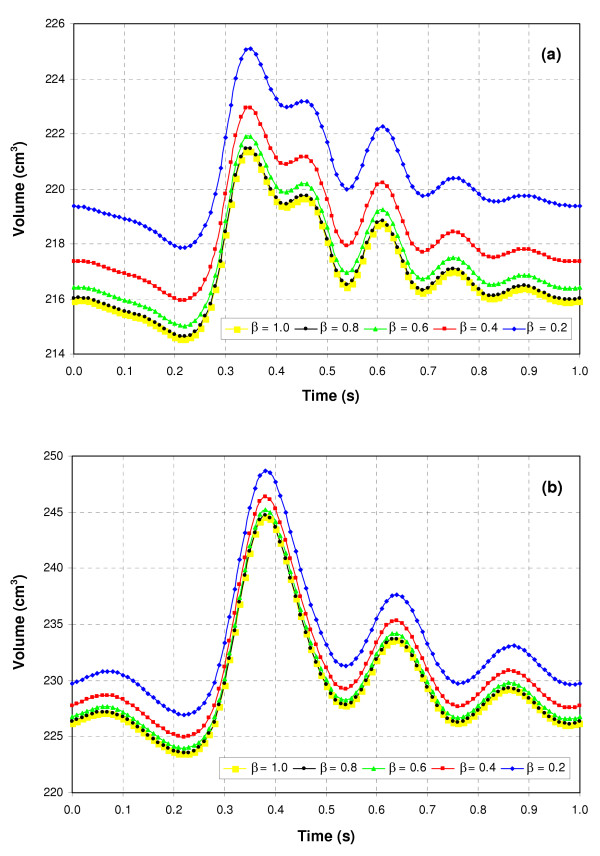
Volume of virtual AAA lumen during the last pulsatile cycle for (a) uniform wall (UW) thickness and (b) variable wall (VW) thickness models.

As evident by Figure 11, the deformations of the AAA sac are not negligible, particularly for a heterogeneous wall. Regardless of asymmetry, the thin midsection of the wall where the diameter and stress are at their maximum, show a significant distortion of the original mesh. Therefore, while the modeling of a heterogeneous wall in this study represents a novel aspect of the research, it must be handled in the computational approach with considerable care so that a stable model can yield accurate and realistic results. The deformation of the geometry is further illustrated in Figure 12, which shows the volume of each virtual AAA's lumen as a function of time for the last cycle of the FSI physics. The initial volume of a non-deformed AAA for all the models used in this study is ∀_o _= 206.6 cm^3^. For the UW models, peak volume is obtained at 0.34 s, while for the VW models, it is at 0.38 s. This coincides with the instant of peak wall stress and peak displacement, at which the AAA wall achieves the greatest expansion. Table 2 shows the change in volume at peak deformation, demonstrating that the increase in volume is concomitant with asymmetry and heterogeneity of the wall. This pulsatile nature of AAA deformation cannot be assessed by utilizing a CSS technique, which takes into account volume changes of the AAA wall only.

**Table 2:** Volume of AAA lumen at peak deformation.

**β**	**0.2**	**0.4**	**0.6**	**0.8**	**1.0**
**UW**	225.1 (+9.0)	222.9 (+7.9)	221.9 (+7.4)	221.5 (+7.2)	221.4 (+7.2)
**VW**	248.7 (+20.4)	246.4 (+19.3)	245.2 (+18.7)	244.7 (+18.4)	244.6 (+18.4)

Peak AAA volume (in cm^3^) and comparison between the UW and VW models. The parentheses show the % increase with respect to the non-deformed geometry volume (∀_o _= 206.6 cm^3^) at the start of the FSI simulation.

### Limitations

Aneurysm rupture does not result solely from the stress exerted along the inner wall, but rather from the transmission of that stress onto the middle and outer wall layers, which causes the diseased arterial wall to fail. Furthermore, it is unlikely that the wall thickness of an actual AAA will decrease concomitantly towards the aneurysm's maximum transverse dimension and then increase by the same gradient towards the iliac bifurcation. The combination of virtual geometry, linearly elastic material properties, and a wall thickness that varies inversely proportional to vessel diameter, creates wall stresses in excess of 100 N/cm^2^, which may be physiologically unrealistic compared to average uniaxial tensile strength of AAA tissue [35]. Nonetheless, our mathematical description of wall thickness variation provides insight into the relative magnitudes of the stresses in UW and VW models, and the importance of wall thickness heterogeneity in the prediction of AAA wall mechanics.

Despite the more accurate predictions of AAA biomechanics utilizing an FSI methodology, there are additional limitations to the present study that restricts its application in a clinical environment. Among these are the assumption of a linear elastic modulus for modeling wall mechanical properties, the need for non-invasive predictors of wall thickness and strength, the lack of inclusion of thrombus and calcification in the geometric and material models, the anisotropic characterization of the tissue models, the absence of external forces induced by surrounding tissue and organs, the absence of iliac arteries, and the lack of assessment of biological activity. Several of these issues are contentious, such as the inclusion of intraluminal thrombus (ILT), as previous authors suggest that ILT may increase or decrease wall stress and aneurysm rupture risk [34,35]. While the investment in computational time proves to be a costly drawback of the FSI methodology with respect to the CSS techniques, the constant improvements in microprocessor technology will allow for practical applications in a clinical setting in the next few years. The ultimate *multi-scale model *for the non-invasive estimation of aneurysm rupture risk should incorporate biomechanical (fluid and solid dynamics), biological, and genetic aspects of AAA disease.

## Conclusion

This work represents a numerical investigation of the fluid-structure interaction of ten virtual abdominal aortic aneurysm models for the prediction of wall stress as a means of assessing rupture potential non-invasively. The effects of asymmetric bulging of the anterior wall and non-uniformity of the wall thickness are studied in detail with respect to the peak wall stress, while maintaining the maximum transverse diameter constant at 6 cm. A comparison is made with traditional numerical techniques based on the quasi-static and transient computational solid stress analyses.

The fluid dynamics in a compliant asymmetric aneurysm model is characterized by the development of ring-shaped vortices during systole that are ejected from the sac shortly after peak pressure is achieved. The distortion energy stored in the vessel as it expands during the cardiac cycle contributes to the early formation of recirculation regions in the aneurysm that yield high velocity gradients at the distal end of the aneurysm. These flow patterns, in combination with the geometrical features of the model and the elastic characterization of the wall material, determine the distribution of flow-induced wall stresses. In a fusiform AAA for which the local thickness decreases inversely proportional to the local vessel diameter, the peak wall stress is 4 times greater than with a uniform wall thickness. Similarly, asymmetric bulging of the anterior wall as determined by β = 0.2 in a uniform wall thickness model results in a 17% increase in peak wall stress when compared to a fusiform AAA. For the same maximum diameter, wall thinning has a more significant effect on the concomitant rise in peak wall stress than the asymmetry of the aneurysm sac. The computational solid stress techniques underestimate wall stress calculations when compared to the fluid-structure interaction predictions. The trade-off for a better predictor tool is a 23-fold increase in computational costs. The use of this numerical technique as a rupture risk assessment tool based on an individual patient's status must incorporate non-invasive predictors of wall thickness and tissue strength.

## Authors' contributions

Alexander Shkolnik created the virtual AAA model, conducted the computational simulations for this study and participated in the methods section of the manuscript. Christine Scotti performed the results post-processing and along with Ender Finol and Satish Muluk contributed to the preparation of the manuscript. Ender Finol also conceived of the study and participated in its design and coordination. All authors read and approved of the manuscript.
